# Supplementary material for: Synthesis and anti-tubercular activity of 3-substituted benzo[b]thiophene-1,1-dioxides
Source: PeerJ. 2014 Oct 7;2:e612. doi: 10.7717/peerj.612 (PMC4193402; doi:10.7717/peerj.612)
Supplement: Supplemental Information 1 — General methods [file peerj-02-612-s001.doc]

**SUPPLEMENTARY MATERIAL**

Synthesis and anti-tubercular activity of 3-substituted benzo[b]thiophene-1,1-dioxides

N. Susantha Chandrasekera, Mai Ann Bailey, Megan Files, Torey Alling, Stephanie K. Florio, Juliane Ollinger, Joshua O. Odingo, and Tanya Parish

TB Discovery Research, Infectious Disease Research Institute, 1616 Eastlake Avenue E, Seattle, WA 98102, USA.

**General Methods**

^1^H and NMR spectral data were recorded in CDCl_3_ or Acetone-d6 on a 300 MHz Bruker NMR spectrometer. Column chromatography was conducted on a Revelaris flash chromatography system. Reactions were monitored using thin-layer chromatography (TLC) on silica gel plates. HPLC analysis was conducted on an Agilent 1100 series LC system (Agilent ChemStation Rev.A.10.02; Phenomenex-Luna-C18, 4.8 mm × 150 mm, 5 μm, 1.0 mL/min, UV 254nm, room temperature) with MeCN/H_2_O (0.05% TFA or HCOOH buffer) gradient elution. HPLC-MS was performed on a Gilson 321 HPLC with detection performed by a Gilson 170 DAD and a Finnigan AQA mass spectrometer operating in electrospray ionisation mode using a Phenomenex Gemini C18 150x4.6mm column. Compounds **3a, b, c, s, t** and **u** were purchased from ChemBridge Corporation.

**Synthesis of 3-bromobenzo[b]thiophene 1,1-dioxide (2)**

To a solution of **1** 1.62 g (7.6 mmol) in 25.0 mL in acetic acid was added 30% aqueous hydrogen peroxide and the mixture was heated for 1 h at 100^o^C. The mixture was poured into ice cold water and let it stand overnight. The resulting solid was filtered and dried to yield **2** (1.65 g, 89%). ^1^H NMR (300 MHz, CDCl_3_): δ 6.98 (s, 1H), 7.58 - 7.72 (m, 4H). LCMS – ESI (M+H)^+^: 214.1.

**General procedure for the synthesis of 3-substituted benzo[b]thiophene-1,1-dioxides**

To a solution of 200 mg (0.82 mmol) of **2** in 5 mL of dimethyl formamide was added 2.0 mmol of the thiol reagent followed by 0.5 mL of triethylamine. The reaction was stirred overnight and washed with 20 mL of deionized water and extracted with 50 mL of ethyl acetate. The organic layer was dried with anhydrous sodium sulfate, filtered and concentrated *in vacuo*. The resulting residue was purified by reveleris flash chromatography system to yield the aryl/heteroaryl thio benzo[b]thiophene 1,1-dioxides.

**3-((5-(4-methoxyphenyl)-1,3,4-oxadiazol-2-yl)thio)benzo[b]thiophene 1,1-dioxide (3d)**

Yield **3d**: (95 mg, 31%). ^1^H NMR (300 MHz, CDCl_3_): 3.9 (3H, OCH_3_. s); 7.0 – 8.0 (m, 9H). LCMS – ESI (M+H)^+^: 373.0.

**3-((5-(4-chlorophenyl)-1,3,4-oxadiazol-2-yl)thio)benzo[b]thiophene 1,1-dioxide (3e)**

Yield **3e**: (122 mg, 39%). ^1^H NMR (300 MHz, Methanol-d_4_): 7.5 – 8.1 (m, 8H). LCMS – ESI (M+H)^+^: 377.0.

**3-(thiazol-2-ylthio)benzo[b]thiophene 1,1-dioxide (3f)**

Yield **3f**: (87 mg, 31%). ^1^H NMR (300 MHz, CDCl_3_): 6.6 (1H, s); 7.6 – 8.1 (m, 6H). LCMS – ESI (M+H)^+^: 282.0.

**3-((4-phenylthiazol-2-yl)thio)benzo[b]thiophene 1,1-dioxide (3g)**

Yield **3g**: (25 mg, 9%). ^1^H NMR (300 MHz, CDCl_3_): 7.7 – 8.0 (m, 10H). LCMS – ESI (M+H)^+^: 358.0.

**3-(benzo[d]thiazol-2-ylthio)benzo[b]thiophene 1,1-dioxide (3h)**

Yield **3h**: (65 mg, 32%). ^1^H NMR (300 MHz, CDCl_3_): 7.3 – 8.1 (m, 9H). LCMS – ESI (M+H)^+^: 332.0.

**3-((5-chloro-3a,7a-dihydrobenzo[d]thiazol-2-yl)thio)benzo[b]thiophene 1,1-dioxide (3i)**

Yield **3i**: (25 mg, 30%). ^1^H NMR (300 MHz, CDCl_3_): 7.4 (1H, s); 7.5 – 8.1 (m, 7H). LCMS – ESI (2M+H_2_O)^+^: 754.9.

**3-((6-ethoxybenzo[d]thiazol-2-yl)thio)benzo[b]thiophene 1,1-dioxide (3j)**

Yield **3j**: (105 mg, 28%). ^1^H NMR (300 MHz, CDCl_3_): 1.5 (3H, d); 4.1 (2H, t); 7.1 – 7.9 (m, 8H). LCMS – ESI (M+H)^+^: 376.0.

**3-((5-methyl-1,3,4-thiadiazol-2-yl)thio)benzo[b]thiophene 1,1-dioxide (3k)**

Yield **3k**: (26 mg, 11%). ^1^H NMR (300 MHz, CDCl_3_): 2.6 (3H, CH_3_, s); 7.7 – 8.0 (m, 9H). LCMS – ESI (M+H)^+^: 297.0.

**3-((5-amino-1,3,4-thiadiazol-2-yl)thio)benzo[b]thiophene 1,1-dioxide (3l)**

Yield **3l**: (16 mg, 7%). ^1^H NMR (300 MHz, CDCl_3_): 6.8 (1H, s); 7.3 (2H, NH_2_, s); 7.6 – 7.8 (m, 4H). Yield **20**: (5 mg, 2%). ^1^H NMR (300 MHz, CDCl_3_): 7.1 (2H, s); 7.6 – 7.8 (m, 4H); 8.8 (1H, SH, s). LCMS – ESI (M+H)^+^: 298.0.

**3-((5-mercapto-1,3,4-thiadiazol-2-yl)amino)benzo[b]thiophene 1,1-dioxide (3m)**

Yield **3m**: (5 mg, 2%). ^1^H NMR (300 MHz, CDCl_3_): 7.1 (2H, s); 7.6 – 7.8 (m, 4H); 8.8 (1H, SH, s). LCMS – ESI (M+H)^+^: 298.0.

**3-((1H-benzo[d]imidazol-2-yl)thio)benzo[b]thiophene 1,1-dioxide (3n)**

Yield **3n**: (100 mg, 39%). ^1^H NMR (300 MHz, CDCl_3_): 6.7 (1H, s); 7.3 – 7.8 (m, 9H). LCMS – ESI (M+H)^+^: 315.0.

**3-((1-methyl-3a,7a-dihydro-1H-benzo[d]imidazol-2-yl)thio)benzo[b]thiophene 1,1-dioxide (3o)**

Yield **3o**: (57 mg, 21%). ^1^H NMR (300 MHz, CDCl_3_): 3.9 (3H, CH_3_. s); 6.4 (1H, s); 7.3 – 7.8 (m, 8H). LCMS – ESI (M+H)^+^: 329.0.

**3-((5-nitro-3a,7a-dihydro-1H-benzo[d]imidazol-2-yl)thio)benzo[b]thiophene 1,1-dioxide (3p)**

Yield **3p**: (142 mg, 48%). ^1^H NMR (300 MHz, CDCl_3_): 7.5 (1H, s); 7.7 – 7.9 (m, 4H); 8.2 (2H, d); 8.5 (1H, s). LCMS – ESI (M+H)^+^: 360.0.

**3-((5-methoxy-3a,7a-dihydro-1H-benzo[d]imidazol-2-yl)thio)benzo[b]thiophene 1,1-dioxide (3q)**

Yield **3q**: (97 mg, 34%). ^1^H NMR (300 MHz, Methanol-d_4_): 3.8 (3H, OCH_3_. s); 6.6 (1H, s); 7.0 – 7.7 (m, 7H).

**3-((1-methyl-1H-tetrazol-5-yl)thio)benzo[b]thiophene 1,1-dioxide (3r)**

Yield **3r**: (115 mg, 50%). ^1^H NMR (300 MHz, CDCl_3_): 4.3 (3H, 3CH_3_, s); 6.9 (1H, s); 7.5 – 8.1 (m, 7H). LCMS – ESI (2M+H)^+^: 561.0.

**3-(pyridin-2-ylthio)benzo[b]thiophene 1,1-dioxide (3v)**

Yield **3v**: (120 mg, 53%). ^1^H NMR (300 MHz, CDCl_3_): 6.6 – 8.5 (m, 9H). LCMS – ESI (M+H)^+^: 276.0.

**3-(pyridin-4-ylthio)benzo[b]thiophene 1,1-dioxide (3w)**

Yield **3w**: (57 mg, 25%). ^1^H NMR (300 MHz, CDCl_3_): 6.6 – 8.5 (m, 9H). LCMS – ESI (M+H)^+^: 276.0.

**3-(isoquinolin-3-ylthio)benzo[b]thiophene 1,1-dioxide (3x)**

Yield **3x**: (95 mg, 36%). ^1^H NMR (300 MHz, CDCl_3_): 7.3 – 8.1 (m, 6H).

**3-(naphthalen-2-ylthio)benzo[b]thiophene 1,1-dioxide (3y)**

Yield **3y**: (110 mg, 42%). ^1^H NMR (300 MHz, CDCl_3_): 5.8 (1H, s); 7.6 – 8.1 (m, 11H). LCMS – ESI (M+2Na)^+^: 671.0.
